# Severe Bone Loss as Part of the Life History Strategy of Bowhead Whales

**DOI:** 10.1371/journal.pone.0156753

**Published:** 2016-06-22

**Authors:** John C. George, Raphaela Stimmelmayr, Robert Suydam, Sharon Usip, Geof Givens, Todd Sformo, J. G. M. Thewissen

**Affiliations:** 1 Department of Wildlife Management, North Slope Borough, Barrow, Alaska, United States of America; 2 Department of Anatomy and Neurobiology, Northeast Ohio Medical University, Rootstown, Ohio, United States of America; 3 Givens Statistical Solutions LLC, Fort Collins, Colorado, United States of America; Texas A&M University, UNITED STATES

## Abstract

The evolution of baleen constituted a major evolutionary change that made it possible for baleen whales to reach enormous body sizes while filter feeding on tiny organisms and migrating over tremendous distances. Bowhead whales (*Balaena mysticetus*) live in the Arctic where the annual cycle of increasing and decreasing ice cover affects their habitat, prey, and migration. During the nursing period, bowheads grow rapidly; but between weaning and approximately year 5, bowhead whales display sustained baleen and head growth while limiting growth in the rest of their bodies. During this period, they withdraw resources from the skeleton, in particular the ribs, which may lose 40% of bone mass. Such dramatic changes in bones of immature mammals are rare, although fossil cetaceans between 40 and 50 million years ago show an array of rib specializations that include bone loss and are usually interpreted as related to buoyancy control.

## Introduction

Bowhead whales (*Balaena mysticetus*) live in the Arctic and subarctic, with some populations migrating north and south with the annual waxing and waning of the sea ice [1,2]. The Western Arctic population, also called the Bering-Chukchi-Beaufort Seas stock, spends much of winter in the Bering Sea, where food intake appears minimal. Conception likely takes place in the Northern Bering/Southern Chukchi Seas, and then whales migrate, feed, and give birth in the Bering, Chukchi and Beaufort Seas until autumn when they migrate south with the expanding winter ice cover [1–3]. Bowheads are baleen whales, and a bilateral rack of approximately 320 baleen plates (640 total) is suspended left and right from the upper jaw [3,4]. It allows them to filter feed on concentrations of tiny prey, such as copepods and euphausiids, and migrate over great distance. The effectiveness of this feeding method is indicated by the large body size (more than 90,000 kg) and great age (more than 150 years) that these animals attain [5].

Bowheads display an unusual growth pattern during the first decade of their life: it has been noted that there is a hiatus in body length growth post weaning, while the head and its baleen keep growing [3,6–9]. This may suggest that resources are limited, or difficult to obtain, and are used for the growth of the food gathering apparatus at the expense of the rest of the body. Baleen plates grow (in length) throughout life, so whales can increase filtering capacity by growth of individual plates and by lengthening the jaws in which the plates are anchored [10].

## Hypothesis

Our life history model indicates that bowhead calves maintain high growth rates and store resources when fed rich milk during nursing, but cannot sustain these rates of growth after weaning because the small size of the baleen rack limits feeding efficiency. The whale’s ontogenetic strategy is to grow the head with its baleen racks at the expense of the rest of the body. Indeed, head and baleen growth may require withdrawal of resources from the rest of the body. Iñupiat whale hunters have noted that ribs of one-year old bowheads are best suited to serve as fishing net weights because they are heavy. This observation inspired our hypothesis: the bone of the ribs of just-weaned, one-year-old bowheads show hyperostosis that occurs neither in fetuses, nor in older whales before sexual maturity. After weaning, young animals with small baleen racks would use bone resources to build the baleen rack, and the ribs would lose their hyperostotic character. Later still, after year 4 or 5, the size of the baleen rack allows the accumulation of sufficient resources from food and body growth starts again.

## Materials and Methods

### Field work

Bowhead whales are harvested by Alaskan Natives (Iñupiat and Yupik), to meet their nutritional and cultural needs, in several villages along the Bering, Chukchi and Beaufort Seas under the auspices of the US Marine Mammal Protection Act, the Endangered Species Act, the NOAA/AEWC (National Oceanic and Atmospheric Administration/Alaska Eskimo Whaling Commission) Cooperative Agreement, and the US membership to the International Whaling Commission (IWC). The Department of Wildlife Management of the North Slope Borough collects data and samples from these whales with the permission of the captains, the Barrow Whaling Captains’ Association, and the AEWC, and reports to the IWC. Our samples were collected under NOAA-NMFS permit No. 17350–00 in Barrow, Alaska.

### Variables studied

#### Whale body dimensions

A standard measuring protocol is used to assess all harvested whales [10]. It includes determining body length, longest baleen plate length, rostrum to blowhole length, and axial girth. Body length is measured from the tip of the rostrum to the midline notch in the tail fluke parallel to the long axis of the whale. Rostrum length is measured from the tip of the rostrum to the middle of the blowholes of the whale, in a straight line, but not parallel to the axis of the whale. Longest baleen length is measured from the distal (ventral) tip of the plate to the end of its keratinized area, which is embedded in the gums of the maxilla. Girth of the whale is determined by measuring a half-circumference at the axilla just posterior to the flipper from the dorsal to the ventral midline, and doubling it. This measure is probably somewhat affected by the position of the whale on the platform, where heavier whales are more deformed by their own weight than smaller specimens. However, the measurement is always taken on the side that is not compressed by the whale’s weight. In addition to these measurements, we also estimated the age of the whales using established methods [6,7,10] that are based on baleen plate length and the isotopic oscillations in these baleen plates that follow the annual migration pattern.

**Fig 1:**
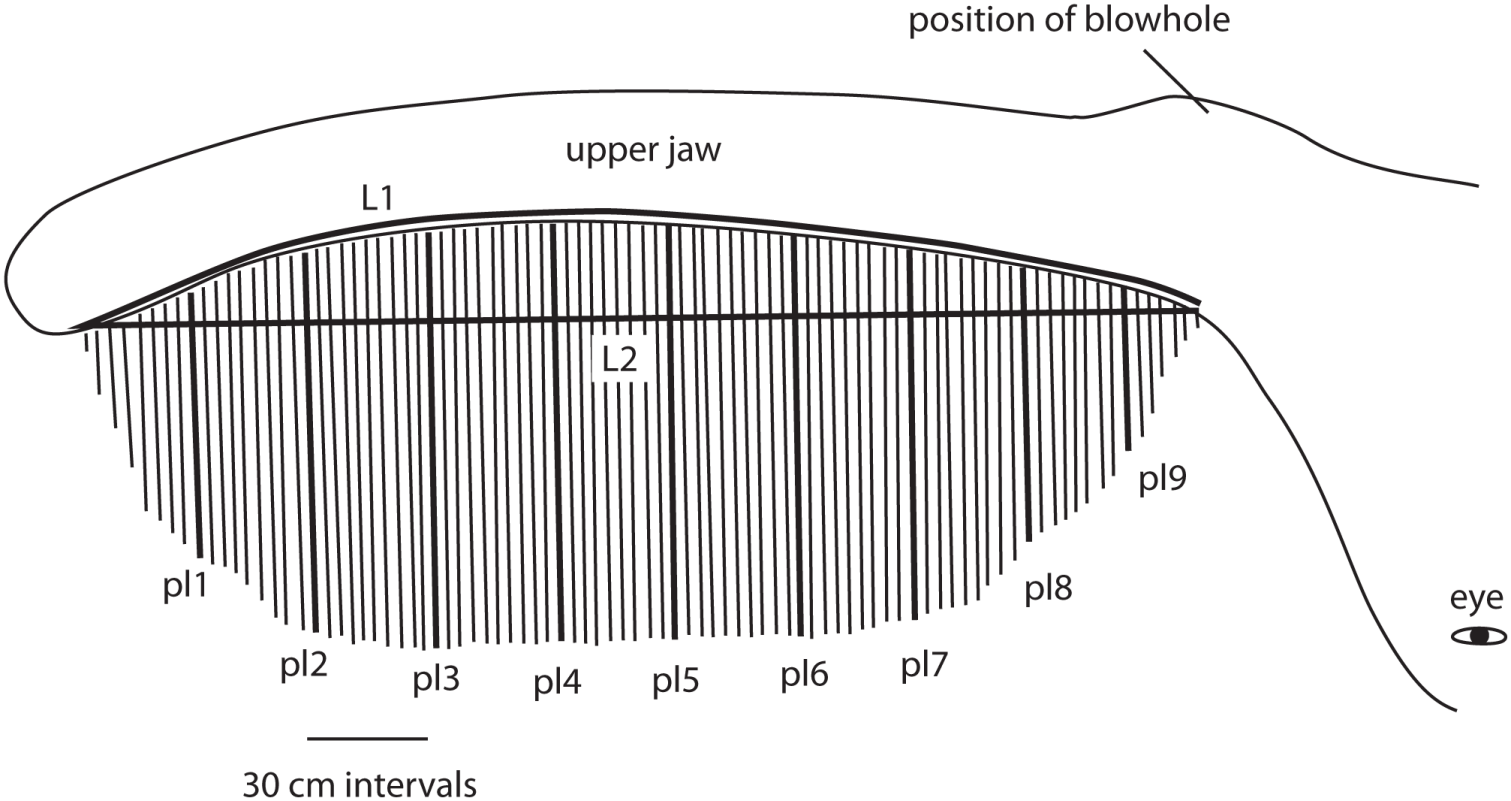
Measurements on baleen rack of bowhead whale. Length of rack was measured along the concave line of implantation of the plates (**L1**) and its chord (**L2**). Baleen plate length was measured at 30 cm intervals along L1, yielding, in this example, nine plate measurements (**pl1**–**pl9**).

As such, our measurements do not include a direct measure of baleen filtering area, so we estimated half the baleen filtering area by multiplying the length of the longest baleen plate with rostrum length. To test whether this estimate indeed correlates with more accurate measurements of the baleen filtering area, we did extensive measurements on one side of the baleen rack of eight bowhead whales (Fig 1), and determined a correlation coefficient between these detailed measurements and our approximation.

For these eight animals, we did not use measurements taken from photographs, as foreshortening of the large racks distorts such measurements, and because individual plates buckle under their own weight when the whale is upside down (its position when accessible for our study). Our method was inspired by that of Kawamura [11], who determined filtering area in sei whales.

We divided the maxilla into 30 cm segments: a jaw with baleen implanted along 290 cm (L1 in Fig 1) would have 9 segments of 30 cm, and one segment of 20 cm. Obviously, using this method, larger whales would have more segments. We obtained measurements of plate length at each 30 cm segment along the upper jaw (pl1-9 in Fig 1; results in S1 File). For all segments, except for the first and last, we modeled the surface area of that segment as a rectangle with length of 30 cm and height of the mean of the plate lengths rostral and caudal to it. Hence, the segment between pl2 and pl3 had an area that equals 30 x (pl2+pl3)/2. We modeled the first segment (30 cm in length) and the last segment (20 cm in length in our example) as triangles. In that example, the surface area of the first segment was 0.5 x 30 x pl1, and of the last 0.5 x 20 x p9. The sum of these two triangles and all the intervening rectangles was then multiplied by the ratio of the linear length between anterior most and posterior most points of the rack (chord, L2) and the length of the (dorsally concave) jaw (L1, 290 in our example), in order to correct for curvature of the jaw.

We followed this procedure in measuring one side of the baleen rack for eight whales of different ages (and thus sizes) within the first two decades of life, the period critical to our study. We then determined whether the product of longest baleen plate and rostrum length (Baleen Filtering Area, BFA, cm^2^) is correlated to the Measured Baleen Area (MBA) as determined by us for the eight whales [12]. The resulting regression equation is:

BFA = 0.9868MBA + 15,255

where the standard error of the estimated slope is 0.1113. The regression relationship is highly statistically significant (F_1,6_ = 78.6, p = 0.0001). Based on the high estimated correlation coefficient between BFA and MBA (R^2^ = 0.9291), we consider BFA to provide a good estimate of filtering area, and use it as a proxy thereof. This allows us to use a database for hundreds of whales where these measurements have been taken in the past, and is generally useful for estimating bowhead baleen filtering area by other researchers. Note that BFA and MBA are measures for area of one side of the baleen rack; this measurement must be doubled to estimate the total filtering area.

#### Rib samples

We removed cross sectional slices of bone approximately 2 cm in thickness from the second rib of bowhead whale individuals with a chainsaw or battery-powered reciprocating saw, in order to study the patterns of change in bone properties with ontogeny. Because we were interested in the function of the ribs as a storage and control mechanism for bone tissue, we sampled the part of the ribs that we considered to be the least likely to be under strong selection for other functions, such as breathing and locomotion. Bowhead ribs have a prominent rugose area for attachment of epaxial muscles just distal to the costal facet, and we took a dorsal sample of the shaft of each rib 20 cm ventral to this area. We took a second slice of each rib 20 cm dorsal to the rib’s ventral (sternal) extremity. The ventral extremity of the rib is widened and grades into cartilage, and is possibly involved in the changes of the shape of the chest during breathing or diving.

We consider the attachment area for the epaxial muscles and the ventral extremity of the rib as homologous points between different individuals, but this implies that the samples taken at 20 cm from these are not homologous if the ribs are different in size. Instead of scaling the position of the slices we used the 20 cm measure for convenience, because samples had to be extracted from a thorax only partly butchered where much of the rib was not visible. Removing a rib from the chest of a bowhead is a slow and precarious process under our field circumstances, and commonly we did not extract entire ribs to take our samples. To test whether the consistent 20 cm distance measurements that were unscaled with regard to body size would disturb the ontogenetic pattern that we were attempting to discern, we extracted complete second ribs for six individuals. For these ribs, we took slices every 20 cm along the entire rib, so as to follow the gradient of change in the variables studied.

### CT-scanning

We used a microCT scanner (Scanco Viva CT 75 Scanner at 70kVp, 114μA, slice thickness of 41 μm) to study several variables, discussed below, of the bone tissue that constitutes the second rib. Larger slices were cut to fit in the scanner and the results averaged based on the proportion they make up of the whole section evaluated. The scanner determines bone density in mg hydroxyapatite per cm^3^ of bone by matching the profile of the sample to a standardized hydroxyapatite phantom. As such, for bone density measurements, all bone in a sample is measured and density is calculated as the mean value of all density measurements.

### Image analysis

**Fig 2:**
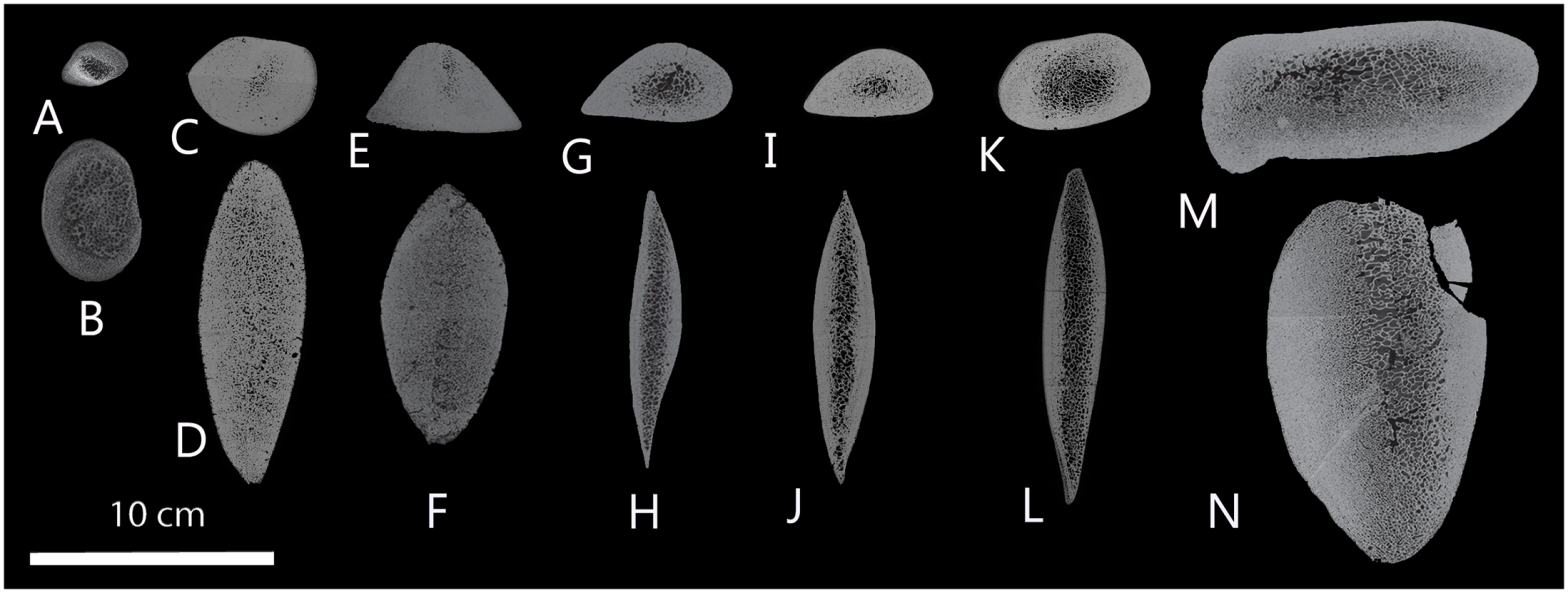
Images of cross-sectioned ribs of bowhead whales. CT-slices of second rib of bowhead whales, taken 20 cm ventral to the muscle scar for the epiaxial muscles (**A, C, E, G, I, K, M**, lateral to top of page, cranial to right), and 20 cm dorsal to the joint for the costal cartilage (**B, D, F, H, J, L, N**, lateral to left of page, cranial to top). Ribs of seven individuals are shown, all to the same scale: a fetus (**A, B**, 2014B6F), two one-year olds (**C, D,** 2015B1; **E, F,** 2014B12), three 2–5 year olds **(G. H,** 2013B7; **I, J,** 2013B6; **K, L,** 2014B15), and a sexually mature individual (**M, N,** 2013B1; crack is damage to specimen).

The images from the scanner were converted to TIFF format and transferred to Avizo 9.0 3D software where a single, cross-sectional slice was extracted (Fig 2). These TIFF images were examined in ImageJ 1.49c, an open source image processing program that was developed at the National Institute of Health. Thresholds for the images followed the isodata method of ImageJ bone analysis software, BoneJ version 1.3.11, which is integrated with ImageJ. We used ImageJ to measure the total cross-sectional areas of each CT slice (Fig 2), as well as the percentage of the total area that was taken up by bone (as opposed to soft tissue between the trabeculae). To visualize trends, we fitted local regression scatterplot smoothing curves to the results (using the loess smoother technique as described in [13]) and implemented in R (R Core Team (2014).

## Results

**Fig 3:**
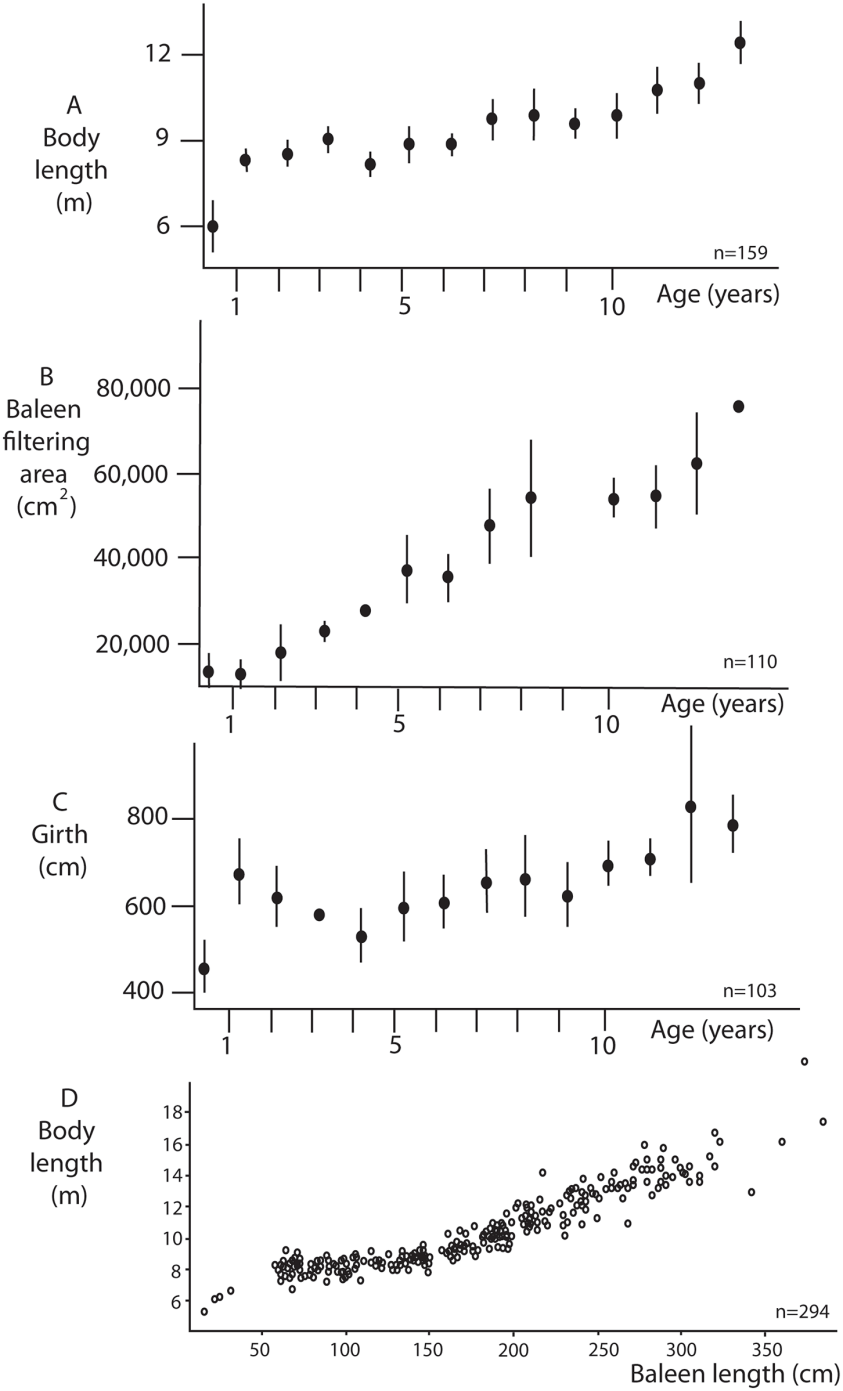
Mean and one standard deviation of body and baleen variables in bowhead whales. Body length (**A**), baleen filtering area (one side) (**B**, the product of rostrum to blowhole length and longest baleen plate length, see method section), and girth (**C**) are plotted against age. A plot of longest baleen plate length against body length (**D**) shows the cessation of body length growth while the baleen plates keep increasing in length. Dots indicate individual data points (in D) or means (in A-C), standard deviations in A-C are indicated by lines.

Bowhead body length and lipid reserves increase rapidly while the calf nurses in the first six to nine months of life [3,7], and blubber makes up approximately 50% of body mass for yearling whales [10]. Our data confirm that, after weaning (~75 cm baleen length), body length all but ceases to increase for several years (Fig 3A [7,10]; see also S2 File). Around age 5, body length increases again and this continues until long after sexual maturity. In spite of the growth hiatus in body length there is no cessation in the growth of baleen (Fig 3B and 3D [10]). Girth decreases in the initial years after weaning (Fig 3C), and increases after that. We assume that the change in girth is mainly due to a loss of adipose tissue. Given that adipose tissue of bowheads is less dense than water [10], we propose that the increase/decrease/increase pattern of girth is offset by a similar pattern in bone, which is far denser than water, and might be used to maintain neutral buoyancy. We found a striking pattern in the ribs.

**Fig 4:**
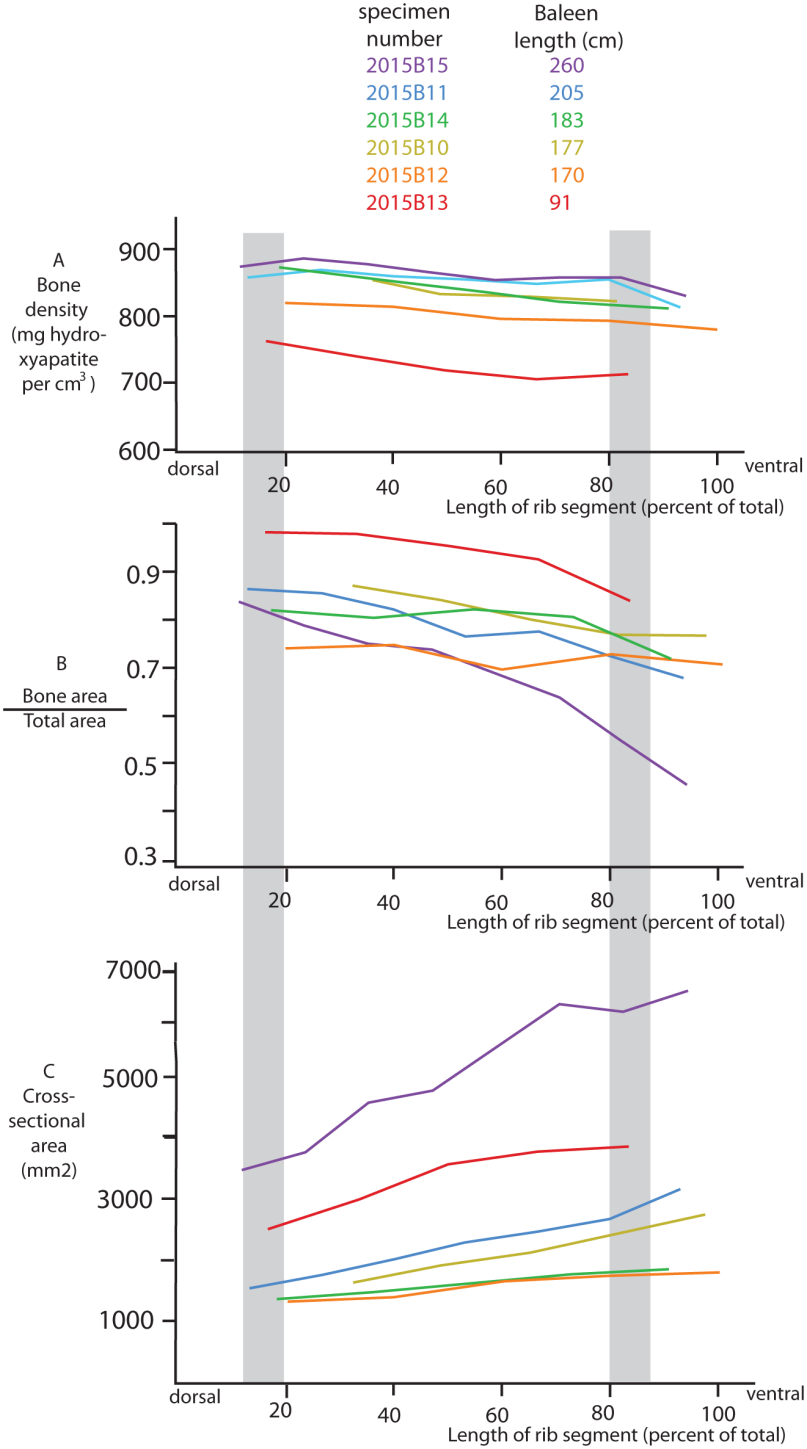
Bone variables of the second rib plotted against its length for six individuals of different ages. The x-axis shows the length of the rib segment from the epaxial muscle attachment (0) to the ventral extremity of the rib (100%). This is plotted against bone density (**A**), the relative amount of bone per total cross-section (as opposed to soft tissue between the trabeculae; **B**), and the total cross-sectional area (**C**). Gray boxes between 0.12–0.20 and 0.80–0.88 indicate the regions were ribs were sampled for the data presented in Fig 5.

Within a single individual, bone tissue of the second rib undergoes a gradual change along a dorsal to ventral trajectory for all investigated age classes (Fig 4; see also S3 File). Density of bone (i.e. the density of a tissue segment consisting solely of bone, excluding marrow space between trabeculae) decreases toward its ventral extremity (Fig 4A). Fig 4B shows the ratio of the rib slice taken up by bone (as opposed to marrow and other soft tissue) divided by the total cross-sectional area. This ratio decreases ventrally as the amount of cortical bone decreases. On the other hand, the total cross-sectional area of the slices increases from dorsal to ventral (Fig 4C).

**Fig 5:**
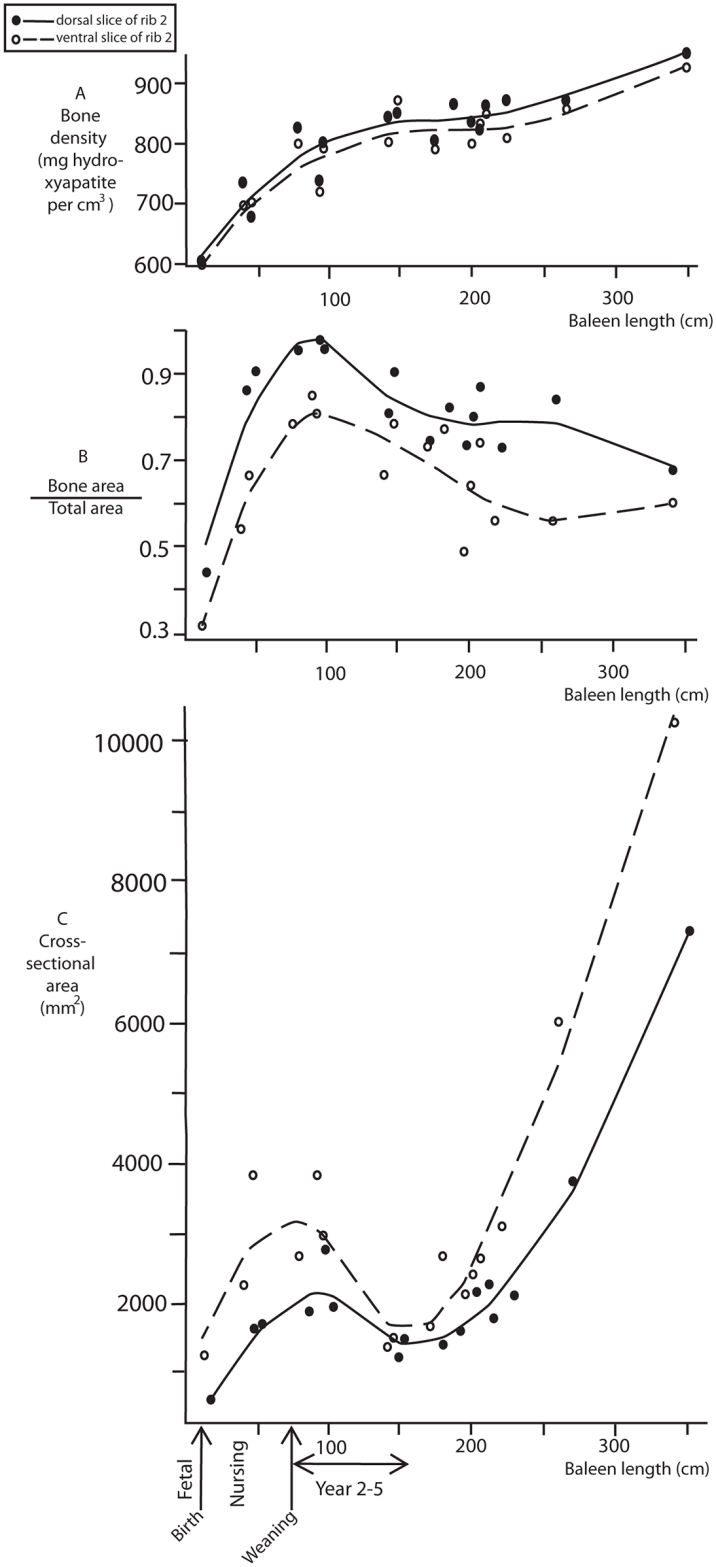
Bone variables of the second rib plotted against greatest baleen length. The x-axis shows baleen length, a correlate of age. It is plotted against bone density (**A**), the relative amount of bone per total cross-section (**B**), and the total cross-sectional area (**C**). Data for dorsal samples and ventral samples are shown, local regression lines were calculated using the loess smoother algorithm [12].

Fig 5 (see data in S3 File) shows results for individuals of different ages, with samples taken 20 cm from the extremities of the rib. If recalculated as a percentage of total rib length, dorsal samples were all taken between 12 and 20% of the length of the rib, and ventral samples between 80 and 88%. These two areas are highlighted by boxes in Fig 4. The samples plotted in Fig 4 indicate that there are no major changes in bone variables in this area, suggesting that our results regarding ontogenetic changes in bone variables as plotted in Fig 5 are not seriously affected by the variability in the exact regions where samples were taken.

Comparing individuals of different ages in Fig 5, our data indicate that bone density is low in whales before weaning (mean = 75 cm baleen length), and then very slowly increases in the first ~ 5 years of life (Fig 5A). This is true for bone density along the entire rib (Fig 4A, where baleen length is a proxy for age). The bone cross-section to total cross-sectional area ratio steeply increases in the first year and then decreases over the next few years by as much as 40% (Fig 5b). Whales in the second year of life have ribs that have barely any spongy bone (Fig 5B). The ratio increases gradually later as the baleen filtering area increases. This trend holds for all segments of the rib, at least for younger whales (Fig 4B, note that we do not have samples along the full length of the rib for older whales).

The cross-sectional area of the ventral part of second rib also changes in ontogeny. Recently weaned whales in their second year of their life have ribs that are massive in diameter ventrally (Figs 2 and 5C), both absolutely, as well as compared to body length, since body length does not increase appreciably over this period. In the years after weaning, rib cross-sectional areas decrease. In fact, the rib cross-sectional area of yearlings is similar to that of adults older than 25 years that are far greater in body size.

## Discussion

### Bowhead Life History

**Fig 6:**
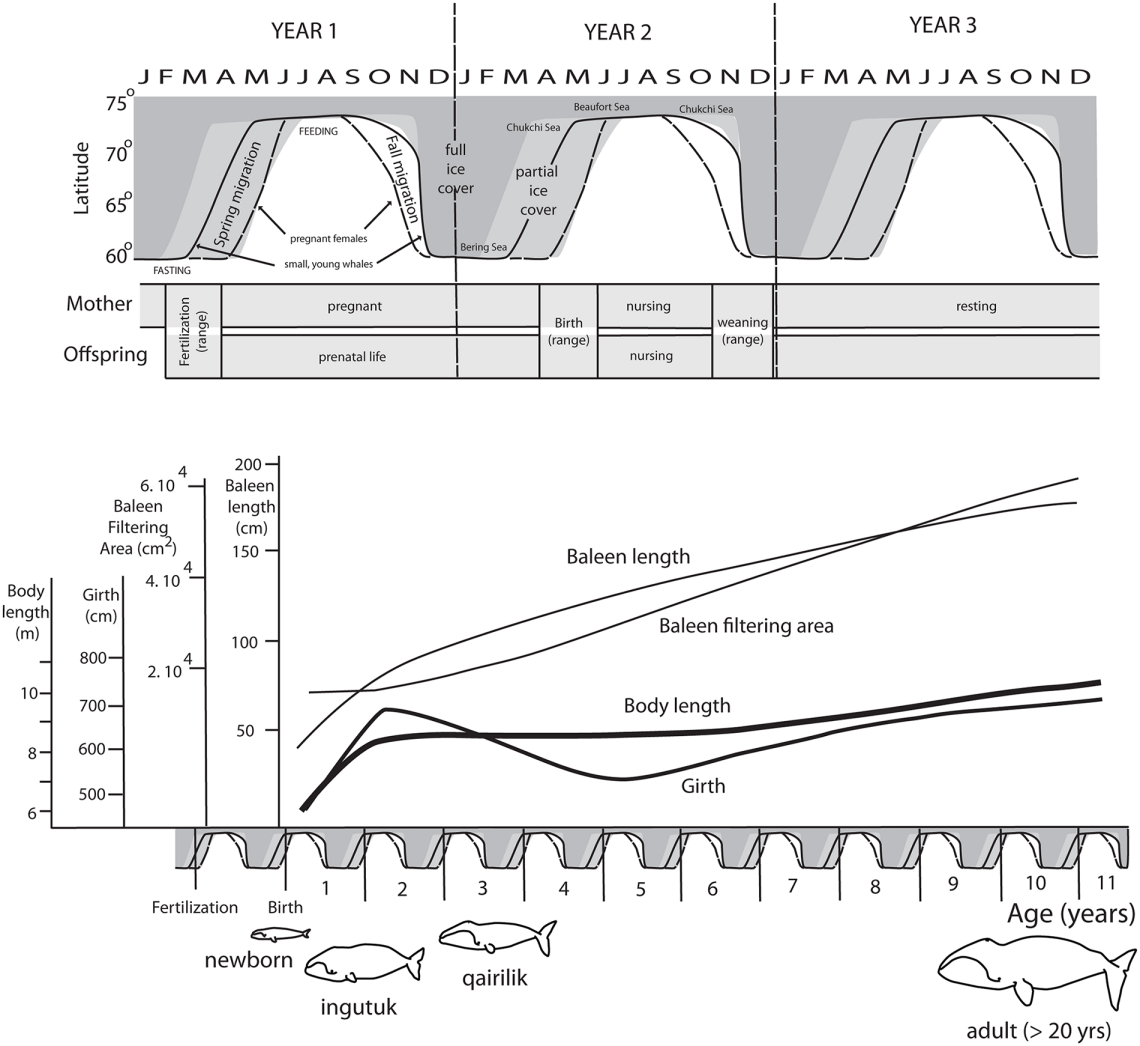
Life history of a bowhead whale. Top panel shows, for a three year period, simplified versions of migration and feeding pattern, ice cover, and reproductive cycle for females and calves. Lower portion shows, for an eleven year period, simplified pattern of morphological variables of whales from birth. Outline drawings show whales at four critical life stages.

The life of a newborn bowhead whale is influenced by the advance and retreat of the sea ice and food availability, just like that of the rest of the stock [1–3, 10]. Birth usually takes place during the northward spring migration. After that, females nurse during the summer feeding season in the Beaufort and Chukchi Seas and may continue nursing through autumn in the Bering Sea. Calves are weaned during the late autumn and early winter ([1–3, 10], Fig 6). Yearlings have thick blubber and sub-dermal fat layers and heavy ribs during their independent northward migration. In subsequent years, blubber layers and rib mass decrease, possibly because the whale’s small baleen rack limits the amount of food that can be procured. We propose that the dense bone provides resources which are needed to build the baleen and the upper jaws in which the baleen plates are anchored. Vertebrates store calcium in their bones, and calcium is an important component of baleen [14]. Both baleen plates and skull grow after weaning, increasing the effective baleen filtering area (Fig 6 [10]), and this growth is at the expense of growth of the rest of the body. In addition to providing resources, catabolism of fat and bone in the chest would affect buoyancy in opposite ways, given that the specific density of these tissues is, respectively, lower and higher than that of water. This may provide a control mechanism for buoyancy.

It is clear that baleen plates do not grow isometrically with body size in the first decade of bowhead life. In fact, the unusual growth pattern of the bowhead head is initiated during the fetal period [15]. This leads to a vastly different body form for whales of different ages, with heads growing disproportionally larger as they age (outlines in Fig 6). The inverse trend occurs in most mammals, where the head grows with negative allometry compared to the body. Indeed, the differences in bowhead body/head proportions of different ages are so large that there was speculation that two species were represented [16]. Alaskan Iñupiat people [17] use the word ‘ingutuk’ to refer to the rotund yearlings [17] and ‘qairilik’ for thin or “tapered” whales of similar length or larger. Yankee whalers called whales that did not yield much oil ‘dry-skins’ some of which may have been qairilik [18].

### Life History of other Mysticetes

Bowhead whales share portions of their arctic feeding grounds with gray whales (*Eschrichtius robustus*), but there is no evidence for a growth hiatus in that species [19]. But then again, gray whales have the shortest and fewest baleen plates of any mysticete [20], and this makes baleen a small investment and may allow yearlings to feed as effectively as adults.

The sister group to bowhead whales are the right whales (*Eubalaena*) which live in temperate and sub-arctic waters. These whales also show a slow-down in body growth and a decrease in blubber thickness in the years immediately after weaning [21–23]. Right whale baleen growth rates are faster than those of bowhead, but the pattern of slowdown of growth with age is similar [6, 21].

### Bone Loss in Mammals

Severe bone loss early in life is very unusual in mammals [24]. In otherwise healthy humans, bone loss can be the result of extreme exercise [25], but also of starvation [26]. In our model, such bone loss is normal in the early stages of a bowhead’s life as baleen and skull grow in order to increase feeding efficiency. Few mammals exploit existing bones to make new bone in a different anatomical region to the extent that bowheads do. Perhaps the best comparison to the bowhead example is found in male cervids, in which the high growth rate of antlers causes temporary physiological osteoporosis [27,28]. Similar to bowhead, bone loss in cervids occurs specifically in non-loaded parts of the skeleton [29], including ribs [30].

Marine vertebrates commonly use bone to control buoyancy [31–34], and the genetic context of some of these adaptations is understood [35]. Changes of bone density with ontogeny occur in plesiosaurs where adults are not pachyosteosclerotic while juveniles are [36]. Among cetaceans, variations in hypo- and hyperostosis have been documented in Eocene species. Such patterns are linked to hunting techniques: fast predators had light bones, whereas ambush predators and bottom walkers had heavy bones [36–40]. In these fossil whales, ribs have been shown to be liable to change, often with dorsal and ventral parts being different morphologically [39–41], reminiscent of the bowhead pattern. The inability of bowheads to obtain sufficient resources for growth may be due to ineffective feeding caused by slow growth of the baleen. This could be the main driver of blubber and bone loss in bowhead. However, the fossil whales studied have teeth, not baleen, and most authors suggest that buoyancy control is the critical factor in their bone loss patterns.

## Conclusions

After weaning, growth in body length all but ceases up to ~ age 5, during which time bowhead whales favor growth of the head and baleen rack over that of the rest of their body, and appear to actively catabolize the ribs to sustain head and baleen growth. Within the lifetime of a single individual, bone mass varies as a result of changing life history requirements. It is possible that such variation in bone mass complicates our ability to recognize variation in life history strategies in morphologically poorly understood modern species. It may also confound our ability to recognize species in the fossil record.

## Supporting Information

S1 File Measurements (Fig 1) used in calculation of baleen rack surface area of bowhead whales used in Fig 3. (XLSX)

S2 File Bowhead whale lengths and baleen plate lengths used in Fig 3. (XLSX)

S3 File Rib bone density data used in Figs 4 and 5. (XLSX)
